# Supplementary figures and images for: Irrespective of Plaque Activity, Multiple Sclerosis Brain Periplaques Exhibit Alterations of Myelin Genes and a TGF-Beta Signature
Source: Int J Mol Sci. 2022 Nov 30;23(23):14993. doi: 10.3390/ijms232314993 (PMC9738407; doi:10.3390/ijms232314993)

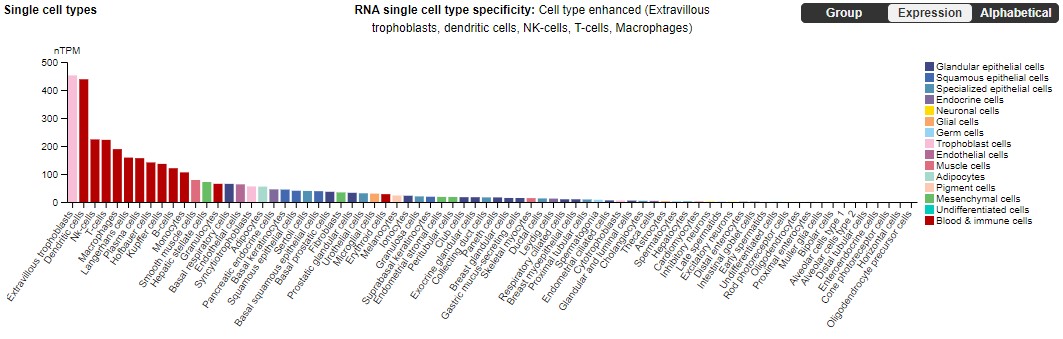

Supplement: Supplementary file 1 [file ijms-23-14993-s001.zip › Figure S1.jpg]

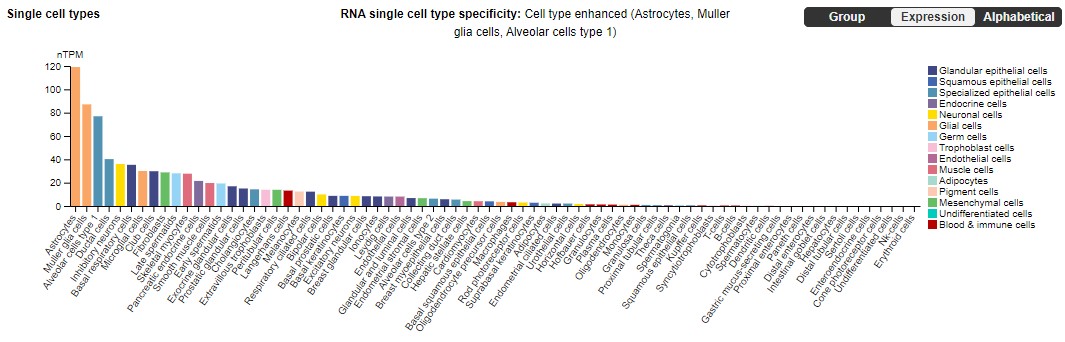

Supplement: Supplementary file 1 [file ijms-23-14993-s001.zip › Figure S2.jpg]
